# Laminin promotes vascular network formation in 3D *in vitro* collagen scaffolds by regulating VEGF uptake

**DOI:** 10.1016/j.yexcr.2014.05.012

**Published:** 2014-09-10

**Authors:** Katerina Stamati, John V. Priestley, Vivek Mudera, Umber Cheema

**Affiliations:** a University College London Tissue Repair and Engineering Centre, Institute of Orthopaedics and Musculoskeletal Sciences, Division of Surgery and Interventional Science, Stanmore Campus HA7 4LP, United Kingdom; b Centre for Neuroscience & Trauma, Blizard Institute of Cell and Molecular Science Barts and The London School of Medicine and Dentistry, 4 Newark Street Whitechapel London E1 2AT, United Kingdom

**Keywords:** Endothelial cells, Angiogenesis, VEGF, Receptors, Integrin, Collagen, Laminin

## Abstract

Angiogenesis is an essential neovascularisation process, which if recapitulated in 3D *in vitro*, will provide better understanding of endothelial cell (EC) behaviour. Various cell types and growth factors are involved, with vascular endothelial growth factor (VEGF) and its receptors VEGFR1 and VEGFR2 key components. We were able to control the aggregation pattern of ECs in 3D collagen hydrogels, by varying the matrix composition and/or having a source of cells signalling angiogenic proteins. These aggregation patterns reflect the different developmental pathways that ECs take to form different sized tubular structures. Cultures with added laminin and thus increased expression of *α*6 integrin showed a significant increase (*p*<0.05) in VEGFR2 positive ECs and increased VEGF uptake. This resulted in the end-to-end network aggregation of ECs. In cultures without laminin and therefore low *α*6 integrin expression, VEGFR2 levels and VEGF uptake were significantly lower (*p*<0.05). These ECs formed contiguous sheets, analogous to the ‘wrapping’ pathway in development. We have identified a key linkage between integrin expression on ECs and their uptake of VEGF, regulated by VEGFR2, resulting in different aggregation patterns in 3D.

## Highlights

• HBMSCs and laminin promote endothelial cell network aggregation.
• Laminin increases endothelial cell VEGF uptake in collagen hydrogels.
• There are more VEGFR2 positive endothelial cells in collagen-laminin hydrogels on day 2 in culture.
• VEGFR1 and VEGFR2 receptors per cell are higher in collagen-laminin cultures than collagen only.

## Introduction

Blood vessels are essential for healthy tissue maintenance as they supply most tissues with oxygen and nutrients [7], [13]. In ischaemic diseases such as peripheral vascular disease and stroke there is compromised blood flow to tissues, with devastating effects to patients [19]. There is an increasing need for grafts and tissue engineered constructs that will mimic blood vessels, and aid rapid vascularisation of the area. Studying and recapitulating parameters involved in neovascularisation *in vivo*, in 3D scaffolds *in vitro*, will increase understanding of EC aggregation and tissue engineering success.

The processes that occur in development to shape blood vessels and other organs in the body into tubes can help our understanding of EC morphology and aggregation. Five processes have been described during different stages in development: wrapping, budding, cavitation, cord hollowing and cell hollowing, as reviewed in Lubarsky and Krasnow [33]. Wrapping involves the movement of an epithelial (or endothelial) sheet outwards, which curls until the ends of the sheet meet to form a tube. In contrast, cord hollowing and cell hollowing involve lumen formation either between cells in a solid structure or within the cytoplasm of a cell that will then fuse with neighbouring cells [33]. Tubes can also form following the elimination of cells from the centre of a larger cylindrical structure (cavitation) or through cell migration from a pre-existing tube (budding) [33].

Angiogenesis involves the formation of new vessels by ECs sprouting and migrating from pre-existing vessels (similar to budding) to an ischaemic area or injury site [17], [32], [38]. Various growth factors are involved in the process, such as VEGF, fibroblast growth factor (FGF) and platelet derived growth factor (PDGF). Although it involves many cell types and growth factors, VEGF is thought to have a significant role [7], [13], [28]. It is especially important in the early stages, by stimulating EC migration and proliferation [38], matrix metalloproteinase (MMP) production and matrix degradation [37].

VEGF activity is affected and regulated by the presence of its ligands or receptors, namely: VEGFR1, VEGFR2, VEGFR3 and co-receptors neuropilin-1 (NRP-1) and NRP2 [22]. VEGFR1 and VEGFR2 are the two main receptors, expressed on most types of ECs [23]. VEGFR2 is the main positive transducer of angiogenesis [7], [40] while VEGFR1 acts as a decoy receptor and inhibitor of VEGF [13]. The type of receptors expressed on ECs can therefore have a significant effect on EC angiogenic response, currently not extensively researched.

*In vitro* studies of angiogenesis commonly use co-cultures of ECs with cells that release angiogenic growth factors such as osteoblasts [43], fibroblasts [41] and HBMSCs [3], [16], [24], [26], [29]. The use of supplementary cells is more physiological than single growth factor addition. The multi-potentiality of the HBMSCs and their high angiogenic growth factor release makes them an ideal cell source for angiogenic studies; therefore they were selected for our study.

We chose collagen I as our scaffold due to its abundance in the human body *in vivo* and its biomimetic characteristics, which make it an ideal scaffold for tissue engineering purposes [5], [8]. We also added basement membrane proteins, laminin and collagen IV, as they are naturally present in the EC environment [2], [1], [21] and can affect EC migration, proliferation and differentiation [14], [21]. Although basement membrane proteins are known to be important in blood vessels, the mechanisms by which they affect EC aggregation are not well understood.

The aim of our study was to test in 3D *in vitro* the role and mechanisms by which HBMSCs and basement membrane proteins affect EC aggregation, as an indicator of EC angiogenic response. The mechanisms by which these parameters affect EC aggregation were tested by correlating with the levels of VEGF present in the different conditions studied, as well as the type and quantity of VEGF receptors (VEGFR1 vs VEGFR2) expressed on ECs, as indicators for anti-angiogenic vs pro-angiogenic response of ECs respectively. To our knowledge this is the first study to test and link these parameters in a 3D *in vitro* environment.

## Methods

### Cell culture

HUVEC׳s were purchased from PromoCell (Germany) and were used between passages 3 and 5. Cells were cultured in Complete Endothelial Cell Growth Media (EGM) (PromoCell, Germany), supplemented with 10% FCS (FirstLink,UK) and 1% Penicillin/Streptomycin (Gibco,UK). HBMSCs were obtained from patients at the RNOH undergoing total hip replacements (with informed consent and ethical approval) and were isolated based on the method by Igarashi et al. [20]. Cells were cultured in low glucose Dulbecco׳s Modified Eagles Medium (DMEM, Sigma USA) supplemented with 20% FCS and 1% Penicillin/Streptomycin. Cells were detached from tissue culture flasks, following washes with Phosphate Buffered Saline (PBS), and incubation with 0.5% trypsin at 37 °C for 5 min.

HUVEC׳s were pre-tested by PromoCell for cell proliferation and morphology. These were also positive for EC specific markers such as CD31 and von Willebrand Factor. HBMSCs were CD31 negative cells, with mesenchymal cell characteristics and differentiation potential into osteoblasts, chondrocytes and adipocytes.

### 3D collagen hydrogel preparation

Collagen type I hydrogels were cast using either HUVECs only (100,000 cells/ml) or with co-cultures of HBMSCs and HUVECs. Four cell ratios were tested by increasing the number of HUVECs in the gels and keeping the number of HBMSCs constant. In all hydrogels 200,000 HBMSCs were used and HUVECs were used between 100,000 cells/ml and 400,000 cells/ml. HBMSC only hydrogels were also cast and used as controls (200,000 cells/ml). Briefly, 800 µl of rat tail collagen type I (2.05 mg/ml, FirstLink, UK) was mixed with 100 µl of 10× Modified Eagle׳s Medium (Gibco, UK ) and was neutralised using drop wise addition of 5 M and 1 M NaOH solution. The cells (in 100 µl medium) were then mixed with the collagen solution and cast in a 12-well plate. Hydrogels were cultured in either EGM (HUVEC only) or in a 1:1 mixture of DMEM and EGM (co-cultures). Endothelial cell only cultures were cultured for 1 week and 2 weeks and co-cultures were cultured for 1 week only (due to excessive contraction of the gels). Hydrogels were analysed for VEGF protein levels using ELISA. VEGF receptors were quantified using flow cytometry and CD31 immunofluorescence was used to test cell morphology and aggregation.

### Basement membrane incorporation into collagen hydrogels

In order to test the effect of basement membrane proteins on endothelial cell morphology, 50 μg/ml of laminin (based on literature) (type V, mouse, BD Biosciences) was added to the cell suspension prior to mixing with the neutralised collagen solution as described above. The effect of collagen type IV (50 μg/ml) (mouse, BD Biosciences) was also tested by mixing with collagen type I prior to neutralization. Basement membrane concentrations were selected based on work by Nicosia et al. [36]. In co-cocultures, the highest number of HUVECs (400,000) was selected as this ratio allowed greater aggregation of cells.

### Integrin *α*6 blocking

In order to test the effect of cell attachment to laminin, 40 μg/ml of anti-integrin *α*6 antibody (as recommended by the manufacturer) (GoH3, Chemicon International) was added to the cultures. Integrins *α*6β1 and *α*6β4 are laminin receptors on ECs and blocking the attachment of ECs to laminin was used to further test its effect on EC morphology.

### Immunofluorescence staining

Hydrogels were fixed in Neutral Buffered Formalin for 1 h and then washed three times for 10 min with PBS. They were then placed in 1% Bovine Serum Albumin (Sigma) (in 0.2% triton/PBS) for 1 h. Following three washes with PBS, the hydrogels were placed in an anti-CD31 mouse anti human monoclonal antibody (1:200 dilution) (Abcam) or an anti VE-cadherin mouse anti human monoclonal antibody (1:200 dilution) (Santa Cruz Biotechnology ) and incubated overnight at 4 °C. The following day the hydrogels were washed three times for 10 min in PBS and incubated with Alexa Fluor 488 chicken anti mouse antibody (Abcam) (1:1000) for 2.5 h. After final washes, the hydrogels were mounted using DAPI mounting medium (Vectashield). Slides were imaged using an upright fluorescence microscope (Olympus BX61) or with a Zeiss LSM710 confocal microscope.

### Image analysis

Images were analysed using the image analysis programme, Image J (NIH, USA), using data from 5–8 fields of view per construct and triplicate samples for each condition. Data was obtained for type of cell morphology, size of cells, length and area of networks and number of nuclei. Cell morphology was defined using the criteria described in Table 1 and percentages were calculated for each type of morphology.

**Table 1:** Criteria used to categorise cell morphologies in cultures.

**Cell type**	**Cell characteristics**	**Size**	**Number of cell processes**	**Endothelial cell cell contact**
	ECs with multiple processes		Multiple	In some cases
	Flattened morphology, large with faint cytoplasmic staining	~1600 μm^2^	None	In some cases
	Flattened morphology, in aggregates	~350μm^2^	None	Yes
	ECs aligned and plasma membrane fused together end to end (>2 cells)	50–250 μm	None	Yes

### Enzyme linked immunosorbent assay (ELISA)

Commercial ELISA kits (R&D systems) were used to test VEGF protein levels in the cultures. On day 7 the supernatant was stored at −80 °C until required. Media samples were assayed from collagen only constructs and collagen with laminin, following the protocol provided in the kit, and the samples were read at 450 nm and corrected at 570 nm *λ*.

### Flow cytometry for VEGFR1 and VEGFR2

Collagen hydrogels were set and cultured for 2 days or 7 days as described above and digested with collagenase type IV (Sigma) at 37 °C for 30 min. Cells were centrifuged, placed in media and incubated at 37 °C on a shaker for 4 h to enable the regeneration of the receptors, as suggested by the manufacturer. 1×10^5^ cells (in 25 µl) were function blocked using mouse IgG (R&D systems) for 15 min at room temperature. Cells were washed and stained separately with 10 µl phycoerythrin (PE) conjugated anti-VEGFR1 or anti-VEGFR2 (R&D systems), as recommended by the manufacturer and published in [23], [22]. Tubes were incubated on ice for 30 min and then washed with stain buffer (PBS 0.5% BSA, EDTA) twice. Cells were re-suspended in 200 µl stain buffer for flow cytometric analysis. In co-cultures, FITC conjugated anti CD31 antibody (BD biosciences) was also used in order to distinguish endothelial cells from HBMSCs, and HBMSC only hydrogels served as negative controls.

Flow cytometry was performed using BD LSRII. Tubes were mixed well before being placed in the flow cytometer and 7 aminoactinomycin D (7 AAD) was added to exclude dead cells. At least 10,000 events were collected for each sample. Cells were firstly gated for live cells and then using a side scatter against PE, the percentage of PE positive cells was calculated. Quantibrite PE beads (BD biosciences) were used in order to convert fluorescence to number of receptors per cell, (supplementary figure) using the same settings as those for cell data acquisition. A linear side to forward scatter plot was used for gating cells and PE beads. Fluorescence geometric means were calculated using FlowJo and using the number of PE molecules/bead provided by the manufacturer, a calibration curve was plotted. The lot specific values for the Quantibrite beads were: high=62,336 molecules/bead, medium-high=23,843 molecules/bead, medium-low=5359 and low=474 molecules/ bead. A curve was plotted for log molecules/bead vs log geometric means. The curve was fitted by linear regression y=mx+b and by solving for *x*=log_10_ (PEmolecules/cell) the number of receptors bound per cell were calculated.

### Statistical analysis

Statistical analysis was performed using Graph Pad software and a two-way ANOVA, a Mann Whitney test, or a t-test were used to test statistical significance, at *p*<0.05. For flow cytometric analysis a Tukey analysis for variance was used.

## Results

**Fig. 1:**
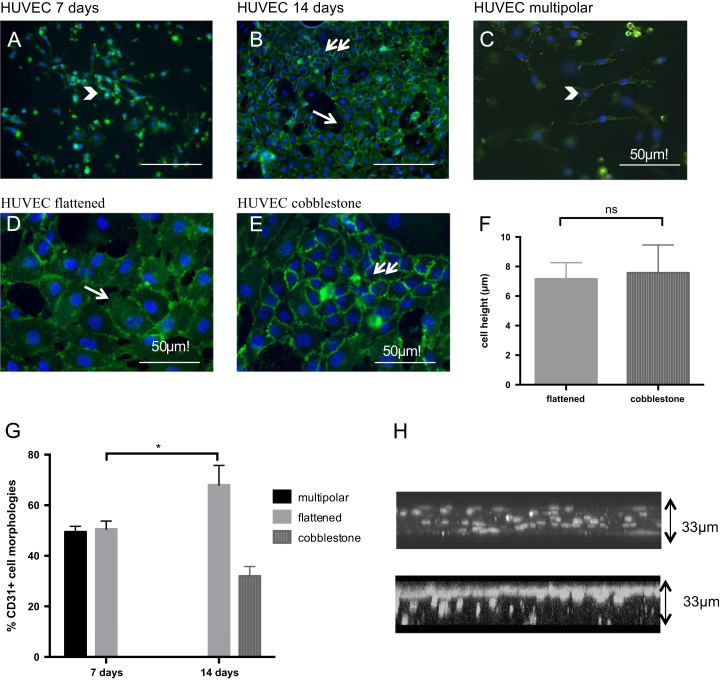
HUVEC only morphologies in collagen hydrogels. A-D CD31 immunofluorescence images showing EC morphologies (CD31=green, DAPI=blue) at day 7 (a) and day 14 (b) (scale bar=100 μm). C-E show higher magnification images of different morphologies, multipolar (C –day 7), flattened (D-day 14), cobblestone (E- day 14) F: Cell height was quantified using confocal images. G: Cell morphologies were quantified at day 7 and 14, ^⁎^*p*<0.05, error bars=s.d. H: Confocal images of the distribution of cells at day 7 (upper panel) and day 14 (bottom panel). Images taken 33 um close to the ventral surface of a 3 mm thick collagen hydrogel.

The aim of our experiments was to test the effect of cell-cell and cell-matrix interactions on EC morphology and aggregation. The hypothesis under test was that co-cultures with HBMSCs would result in distinct EC morphologies compared to EC only cultures. Table 1 shows the different morphologies of ECs observed in 3D collagen hydrogels, and the criteria used to classify them. Four different morphologies were observed, namely: 1) multipolar, 2) flattened, 3) cobblestone, and 4) networks. Briefly, multipolar cells were spindle-like cells with multiple cell processes extending from them. Flattened cells were usually polygonal in shape and formed some aggregates. Cobblestone cells were smaller cells than the flattened and formed clusters or aggregates (Table 1, Fig. 1). Networks were EC end-to-end aggregates. A major component, which defined these different morphologies, was the (2D) size of the cell, which was significantly different (*p*<0.01) between the flattened (1600 μm^2^) and cobblestone cells (345 μm^2^) (Table 1). The height or thickness of the cells, as measured by confocal microscopy, was the same between the flattened (7.16 μm) and cobblestone morphologies (7.57 μm) (Fig. 1F).

### Endothelial cell morphology in 3D collagen hydrogels

Initial experiments involved culturing ECs in collagen hydrogels without the addition of any supplementary cells or extracellular matrix proteins to test the morphological progression of ECs. There were multipolar, flattened and cobblestone cells in EC only cultures. As seen in Fig. 1, the progression from multipolar to flattened to cobblestone morphology was time-dependent. As culture time increased, EC migration within the collagen hydrogels was evident, with the majority of the cells at the end of the two-week culture period found on the ventral surface of the scaffold. Cell migration was initially seen using a normal upright microscope and confirmed using confocal microscopy. As cells migrated to the top of the ~3 mm deep scaffold a cobblestone-flattened cell sheet in 3D formed. This cell sheet was mainly found on the 33 μm ventral aspect of the constructs. *Z* stack images of EC only collagen constructs on day 7 showed cell nuclei interspersed throughout the hydrogel. In contrast, on day 14 cell nuclei were primarily present on the ventral surface of the scaffold (Fig. 1H).

**Fig. 2:**
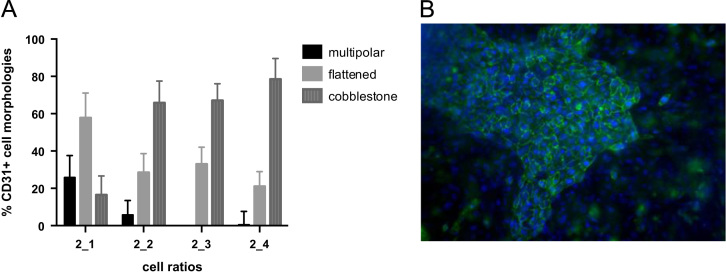
HUVEC morphologies in co-cultures with HBMSCs. A. Percentage of CD31 positive EC types using different ratios of cells. Error bars=s.e.m, B. CD31 immunofluorescent image of a 2:4 co-culture at day 7 (CD31=green, DAPI=blue) (scale bar=100 µm).

The presence of HBMSCs within the culture resulted in the formation of the cobblestone morphology (cell-cell interactions) within 7 days- compared to ECs only where cobblestone formation was mainly evident at 2 weeks (Fig. 2a,b). Different cell ratios were tested, and EC morphologies were quantified (Fig. 2a). The aggregates that formed in the highest EC-HBMSC cultures (2:4 ratio) were larger in terms of surface area occupied than in the other ratios (data not shown). This ratio was selected for all other studies as it provided adequate growth factors, measured using ELISA, and allowed for higher cell aggregation.

### Cell matrix interactions alter endothelial cell aggregation patterns

Endothelial cells are usually in contact with basement membrane proteins in vivo, which include laminin and collagen type IV. The aim of these experiments was to test the effect of cell-matrix interactions on EC-EC aggregation patterns. We tested the morphology of the cells in the presence of basement membrane proteins by incorporating laminin or collagen IV in type I collagen hydrogels of EC only cultures and co-cultures.

**Fig. 3:**
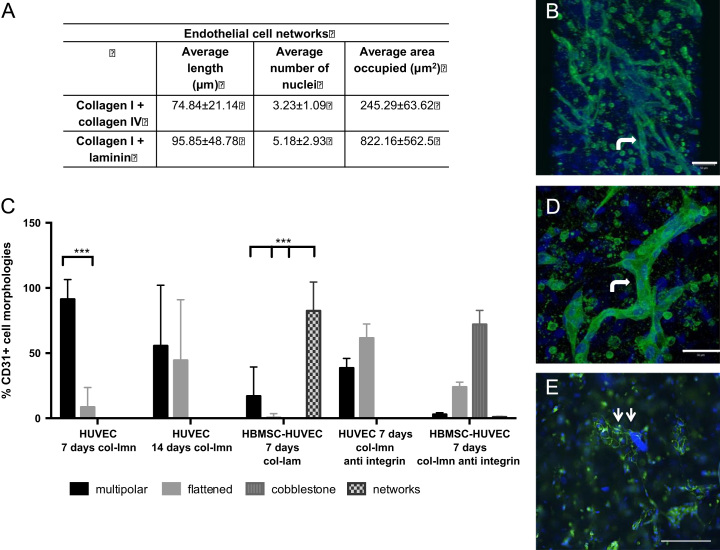
EC network aggregation in collagen hydrogels with basement membrane proteins. A) Table showing the average length, number of nuclei and area occupied by the network aggregates in collagen hydrogels with either collagen IV or laminin B) Confocal image of network aggregates in co-cultures with laminin (bent arrow) C) graph shows the percentage of cell types in cultures with laminin and with anti integrin antibody, D) Higher magnification confocal micrograph image of an end-to-end network in co-cultures with laminin (bent arrow) E) Fluorescent micrograph of anti-integrin experiments with co-cultures where cobblestone aggregates were seen (two arrows) (scale bar=100 μm) (CD31=green, DAPI=blue).

**Fig. 4:**
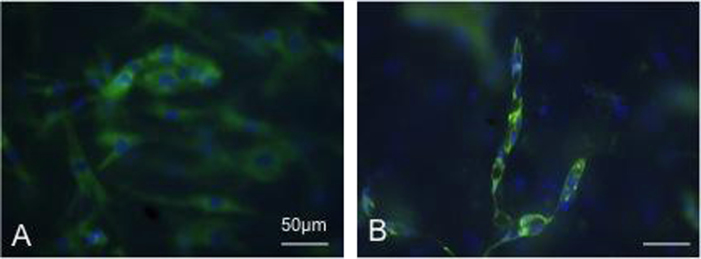
VE-cadherin immunofluorescence staining. A. collagen only co-cultures, B. co-cultures in collagen-laminin constructs. Scale bar=50 μm, (VE-cadherin=green, DAPI=blue).

EC morphological progression was altered in the presence of laminin (Fig. 3). The addition of laminin in the co-cultures resulted in EC aggregation in end- to-end networks (Fig. 3). ECs fused together to form the networks, in contrast to the cobblestone aggregation in the collagen only hydrogel co-cultures (Fig. 2). There were no cobblestone aggregates in collagen-laminin co-cultures (Fig. 3). VE-cadherin staining also showed intense membrane staining in co-cultures with laminin, (Fig. 4) indicating EC cell-cell communication. In collagen only co-cultures there was no membrane localisation of VE-cadherin by day 7. Instead, staining was more prominent in the cytoplasm of the cells.

We also tested the effect of collagen type IV on EC morphology (Fig. 3). Addition of collagen type IV to collagen I showed that laminin is a more potent stimulus for network formation (Fig. 3). On average, networks with collagen IV were around 74 μm in length, (ranged 40–120 μm) whereas with laminin approximately 95 μm (50–250 μm length) (*p*<0.05). Networks also occupied four times the surface area in collagen-laminin (822 μm^2^) hydrogels than in the collagen IV and I hydrogels (245 μm^2^). The data suggested that laminin induced a faster and greater change in EC aggregation.

**Fig. 5:**
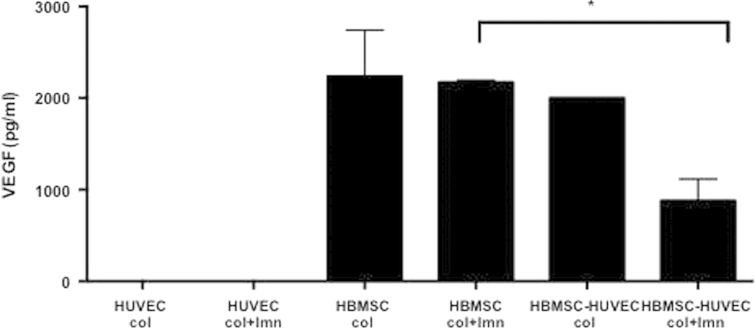
VEGF concentration in media samples of cultures with EC only, HBMSC only and co-cultures with or without the added laminin, ^⁎^*p*<0.05, error bars- s.d.

ECs aggregated into networks in cultures containing laminin, only where HBMSCs were present indicating that a source of angiogenic growth factors (measured in HBMSC constructs- Fig. 5) was necessary for this cell aggregation [3], [16], [24], [26], [29]. We found that increasing the number of HUVECs in monocultures to equal the total number of cells in co-cultures (600,000 cells), thus increasing cell-cell contact, did not enhance network aggregation (data not shown). This further suggested that network formation was not a direct result of EC-EC contact but that HBMSCs were necessary.

### Prevention of EC-laminin attachment via integrin *α*6 expression prevents end-to-end aggregation

In order to test the effect of cell attachment to laminin and confirm the changes in EC morphology, we added an integrin *α*6 antibody to collagen- laminin hydrogels. Integrins *α*6β1 and *α*6β4 are involved in the attachment of ECs to laminin (Primo et al. 2010). Blocking *α*6 integrin resulted in 46% of the ECs in the HUVEC only cultures showing the “flattened” morphology (Fig. 3c), similar to the percentages (55.6%) seen in collagen only cultures (Fig. 1g). Cells in co-cultures aggregated in the cobblestone morphology (72%) as seen in Fig. 3c, similar to results with collagen only co-cultures (78%, Fig. 2a). The end-to-end, or tubular network aggregation observed when ECs attached to laminin via the *α*6 integrin was eliminated where the integrin attachment was blocked (Fig. 3).

### VEGF protein production in cultures was dependent upon the presence of HBMSCs

Angiogenic growth factors are critical during different stages of angiogenesis in vivo. We tested the hypothesis that HBMSCs would be producing growth factors required to stimulate morphological changes in ECs for early capillary formation and aggregation. We specifically tested VEGF levels (as an exemplar angiogenic growth factor). ELISA was used to quantify VEGF levels in the media of hydrogels with and without laminin. HUVECs did not produce any detectable levels of VEGF in either collagen only or collagen-laminin cultures. There was a significant (*p*<0.05) decrease in the amount of VEGF in the medium of laminin co-cultures (880 pg/ml) compared to collagen only hydrogels (2000 pg/ml) (Fig. 5). This was postulated to either mean VEGF was being trapped in collagen-laminin constructs, or more VEGF was uptaken by ECs in the collagen-laminin constructs. We tested both of these factors.

There was no significant difference in VEGF levels in HBMSC only cultures with or without laminin, which suggests that laminin did not directly affect the production of VEGF by HBMSCs or indeed that the laminin-collagen constructs trapped any of the VEGF. VEGF receptors were therefore quantified in order to test VEGF uptake by ECs.

### Enhanced VEGFR2 receptors on ECs where *α*6 integrin mediated laminin attachment Is present

VEGFR1 and VEGFR2 receptors were quantified using flow cytometry. We specifically tested the number of cells positive for VEGFR1 and VEGFR2 and the average number of receptors per cell in collagen constructs compared to collagen- laminin constructs.

**Fig. 6:**
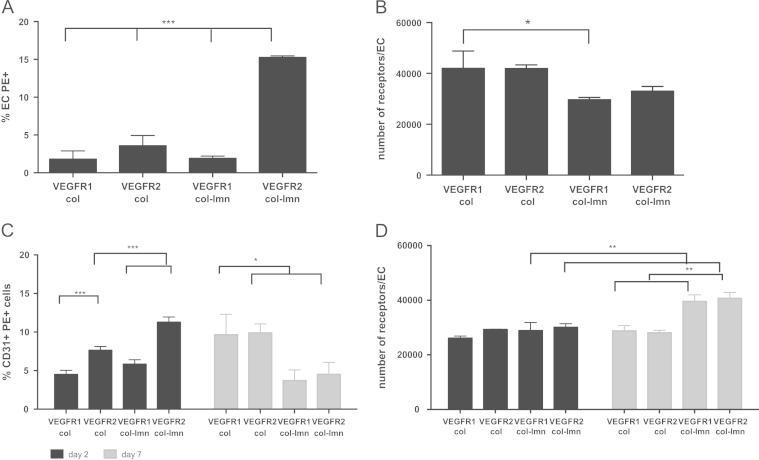
Flow cytometry analysis of VEGFR1 and VEGFR2 in EC only cultures (A,B) and co-cultures with HBMSCs (C,D). A, C: Percentage of PE positive ECs, B,D: number of receptors per cell as calculated by the use of PE beads. Error bars=s.d., significance ^⁎^*p*<0.05, ^⁎⁎^*p*<0.01, ^⁎⁎⁎^*p*<0.001.

As shown in Fig. 6, in EC only cultures there were significantly (*p*<0.001) more VEGFR2 positive cells (~15%) in collagen-laminin cultures (with activation of the *α*6β1 and *α*6β4 integrins) than collagen only cultures (~4%) (Fig 6a). There were also significantly (*p*<0.001) more VEGFR2 positive cells in collagen-laminin (~15%) cultures than VEGFR1 (~2%) cells in EC only cultures. When the number of receptors per cell was analysed, we found that collagen only cultures had significantly (*p*<0.05) more VEGFR1 receptors per cell than laminin cultures (Fig 6b).

In co-cultures, on day 2, the number of cells positive for the receptors and CD31, i.e. ECs, for VEGFR2 was significantly higher (*p*<0.001) in collagen-laminin (~11%) cultures compared to collagen only (~7%) (Fig 6c). There were also significantly (*p*<0.001) more VEGFR2 positive cells than VEGFR1 positive cells in either collagen only cultures (~7% to ~4%) or collagen-laminin cultures (~11% to ~6%). On day 7, the number of cells positive for both receptor types decreased in laminin cultures but increased in collagen only. The number of cells positive for bothVEGFR1 and VEGFR2 was significantly lower (*p*<0.05) in laminin cultures at this time point than collagen only cultures.

The number of receptors per cell was also analysed and no differences were found between the two culture conditions and the receptor types at day 2 (Fig 6d). On day 7 however there was a significant increase (*p*<0.01) in the number of receptors per cell for both VEGFR1 and VEGFR2 in collagen-laminin cultures compared to day 2. There were also significantly (*p*<0.001) more receptors, both VEGFR1 and VEGFR2, in collagen-laminin cultures than collagen only cultures.

## Discussion

Our study tested the effect of matrix composition, thus cell-matrix interaction and cell-cell interactions on EC aggregation. Specifically we were able to correlate morphological aggregation patterns with cell-matrix attachment via integrins and VEGF receptor upregulation. We have shown for the first time a link between matrix composition, specifically *α*6 integrin expression, critical for cell-laminin attachment, and increased VEGFR2 receptor expression in ECs within a 3D *in vitro* setting. The types of receptors present were quantified and the effect of matrix composition and the HBMSCs on the number and type of VEGF receptors expressed on ECs were also tested.

While many studies use ECs seeded on the surface of 3D scaffolds, including collagen, in the current study ECs and HBMSCs were interspersed throughout the matrix. This mimicked more closely the 3D environment found in vivo. Several studies using collagen hydrogels use Phorbol Myristate Acetate (PMA) and growth factor supplemented media [11]. The presence of these factors results in EC tube formation [4], [11], [9]. In this study however we relied on the effect of supplementary cells and laminin to induce EC morphological and aggregation changes.

Laminin and HBMSCs promoted end-to-end network formation in co-cultures, inhibiting the cobblestone sheet formation, which was the predominate morphology in collagen only cultures. The EC cobblestone sheet that was found in collagen only cultures could be compared to the “wrapping” process in development. This is further supported by migration of the cells in EC only cultures to the surface of the constructs as cells aggregate into cobblestone. On the contrary, ECs in collagen-laminin co-cultures with HBMSCs showed morphologies that could be mimicking cavitation, cord hollowing or cell hollowing. However, the presence of hollow lumens has not been determined in this study. Future studies will aim to recapitulate these developmental processes more accurately within our 3D scaffolds.

Laminin is known to be important in promoting EC network aggregation, especially in matrigel. Blocking laminin in matrigel experiments inhibits endothelial cell differentiation and tube morphogenesis [10], [18]. Although matrigel is widely used in angiogenesis studies, it has disadvantages compared to collagen, such as its predetermined composition and its ability to induce cell fusion and tubulogenesis in cells such as fibroblasts [12]. Collagen IV also promoted network aggregation in our study, however not as extensively as laminin, in agreement with the results of other studies [30], [35], [36]. We therefore chose to focus on the use of laminin in our cultures.

Similar VEGF levels were produced in HBMSC only cultures with and without laminin. VEGF level similarities suggest that laminin does not affect HBMSC signalling and that the addition of laminin to collagen hydrogels does not trap extra VEGF. However, VEGF levels were significantly lower in collagen-laminin co-cultures compared to collagen only co-cultures. This suggested higher VEGF uptake by ECs in these conditions, which was supported by VEGF receptor number quantification. Future work will test the addition of fluorescently labelled VEGF in culture medium, which can be tracked and quantified within cells, similar to work by others [34].

The presence of VEGF in co-cultures would result in 50% of the receptors being internalised within 10 min of exposure to the protein [15], [23], [39]. This fast internalisation would result in quick initiation of signalling cascades [34]. The number of positive cells for bothVEGFR1 and VEGFR2 was lower on day 7 compared to day 2 in collagen-laminin co-cultures. This suggests that a proportion of ECs no longer expressed the receptors as time in culture progressed and cell aggregation patterns changed. At the same time, data for the number of receptors per cell show an increase, which could be attributed to receptor stabilisation on those cells that continue to positively express these receptors.

VEGFR2 positive cells in collagen-laminin co-cultures decreased more than VEGFR1. This suggests higher VEGFR2 internalisation compared to VEGFR1 and a greater R2 homodimer formation than R1/R2 heterodimer formation. This is significant because VEGFR2 homodimer formation results in a stronger pro-angiogenic signal [7], [40].

VE-cadherin has been shown to be important for EC tube formation and blocking its activity inhibits tube formation (Bach et al. 1998, Yang et al. 1999). Qualitative evidence through immunostaining in this study showed that VE-cadherin surface localisation on day 7 was more prominent in our collagen-laminin co-cultures compared to collagen only (Fig. 4). Higher VE-cadherin levels have previously been shown to correlate with higher VEGFR2 expression [6], [31], [34], [39]. VEGFR2 surface expression is stabilised as a result of the interaction with cadherins, decreasing receptor internalisation [6], [31], [34], [39]. Further quantitative evidence will be needed to determine whether VE cadherin affected receptor internalisation or stabilisation in collagen-laminin cultures.

While our work quantified VEGF receptor expression on ECs cultured within a 3D culture environment, published work is based on 2D culture of cells [22], [23]. The surface levels of both receptor types were much higher than previously published work [23], [22] using similar quantitation techniques. Their work however used *in vitro* 2D culture of ECs or *ex vivo* cultures. In fact, results comparing receptor levels on ECs in *ex vivo* cultures and *in vitro* cultures had shown significant differences [23]. This emphasises the importance of cell matrix interactions for VEGF receptor expression. In the current study we hypothesise that both the 3D environment and the presence of VEGF-releasing HBMSCs, resulted in increased VEGF receptor expression.

Although we tested only VEGF levels within our cultures, others (and our group, unpublished data) have shown that HBMSCs produce a variety of different factors *in vitro* such as bFGF, angiopoietin-1 and interleukins [27], [29]. We hypothesise that HMBSCs acted primarily as a growth factor factory, providing ECs with a cocktail of angiogenic growth factors [3], [16], [25], [26], [29]. Additional factors such as MMPs, collagens and laminins, not tested in this study, influence EC morphology and aggregation and can be produced by both cell types used ([19], [42]).

## Footnotes

Appendix A Supplementary data associated with this article can be found in the online version at 10.1016/j.yexcr.2014.05.012.
