# Supplementary material for: Laminin promotes vascular network formation in 3D in vitro collagen scaffolds by regulating VEGF uptake
Source: Exp Cell Res. 2014 Sep 10;327(1):68–77. doi: 10.1016/j.yexcr.2014.05.012 (PMC4155934; doi:10.1016/j.yexcr.2014.05.012)
Supplement: Supplementary file 1 — Supplementary Material [file mmc1.pptx]

## Slide 1
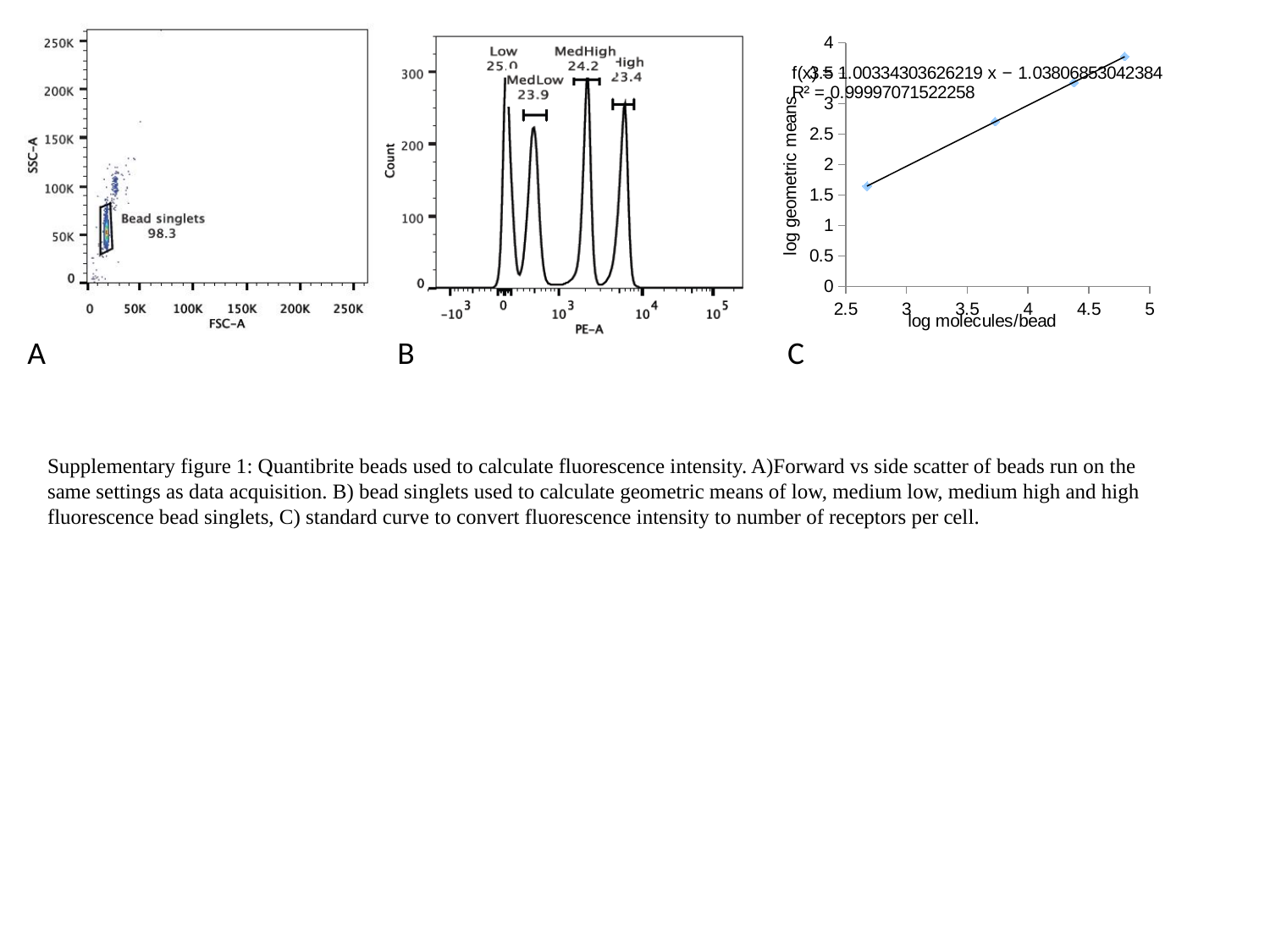

### Chart
| Category | |
|---|---|A
B
C
Supplementary figure 1: Quantibrite beads used to calculate fluorescence intensity. A)Forward vs side scatter of beads run on the same settings as data acquisition. B) bead singlets used to calculate geometric means of low, medium low, medium high and high fluorescence bead singlets, C) standard curve to convert fluorescence intensity to number of receptors per cell.
